# Extent and structure of health insurance expenditures for complementary and alternative medicine in Swiss primary care

**DOI:** 10.1186/1472-6963-6-132

**Published:** 2006-10-11

**Authors:** Andre Busato, Reiner Eichenberger, Beat Künzi

**Affiliations:** 1 Institute for Evaluative Research in Orthopaedic Surgery, University of Bern, Stauffacherstrasse 78, CH-3014 Bern, Switzerland; 2 Seminar für Finanzwissenschaften, Pérolles 90, CH-1700 Freiburg, University of Freiburg, Switzerland; 3 Swisspep – Institut für Qualität und Forschung im Gesundheitswesen, Postfach – CH 3073 Guemligen, Switzerland

## Abstract

**Background:**

The study is part of a nationwide evaluation of complementary and alternative medicine (CAM) in primary care in Switzerland. The goal was to evaluate the extent and structure of basic health insurance expenditures for complementary and alternative medicine in Swiss primary care.

**Methods:**

The study was designed as a cross-sectional evaluation of Swiss primary care providers and included 262 certified CAM physicians, 151 noncertified CAM physicians and 172 conventional physicians. The study was based on data from a mailed questionnaire and on reimbursement information obtained from health insurers. It was therefore purely observational, without interference into diagnostic and therapeutic procedures applied or prescribed by physicians. Main outcome measures included average reimbursed costs per patient, structured into consultation- and medication-related costs, and referred costs.

**Results:**

Total average reimbursed cost per patient did not differ between CAM physicians and conventional practitioners, but considerable differences were observed in cost structure. The proportions of reimbursed costs for consultation time were 56% for certified CAM, 41% for noncertified CAM physicians and 40% for conventional physicians; medication costs – including expenditures for prescriptions and directly dispensed drugs – respectively accounted for 35%, 18%, and 51% of costs.

**Conclusion:**

The results indicate no significant difference for overall treatment cost per patient between CAM and COM primary care in Switzerland. However, CAM physicians treat lower numbers of patients and a more cost-favourable patient population than conventional physicians. Differences in cost structure reflect more patient-centred and individualized treatment modalities of CAM physicians.

## Background

The regulation of health insurance coverage for complementary and alternative medicine (CAM) varies considerably across different national health plans. Increased use of CAM and consistent lobbying from CAM practitioner and health consumer groups has increased pressure upon policy makers to include CAM in basic health coverage in various countries. Following a political discussion, the Swiss Federal Department of Home Affairs decided in 1998 to add five methods of complementary medicine to the benefit package of basic health insurance for a pilot period of five years. The methods included homeopathy, anthroposophical medicine, neural therapy, Western herbal medicine (phytotherapy), and traditional Chinese herbal medicine. Separately, acupuncture was included on a permanent basis in the same context based on a positive appraisal of the Swiss federal expert commission for health insurance benefits. Reimbursements of expenditures for alternative medicine were covered by the basic health insurance package only when these methods were provided by physicians with appropriate CAM training approved by the Swiss Medical Association. Because of the probationary nature of including CAM procedures in health plans, a nationwide evaluation of CAM – including several studies aiming at efficacy and cost efficiency – was performed. Based on the results of this evaluation [1] the Swiss Federal Office of Home Affairs decided in 2005 to withdraw CAM procedures (but not acupuncture) from basic health insurance coverage.

As part of nationwide evaluation, the goal of this study was to evaluate the amount and structure of health insurance expenditures of certified CAM physicians and to compare them with physicians providing conventional primary care in Switzerland. The literature provides some empirical evidence that CAM may reduce treatment and referral costs in primary care[2]. Therefore the specific research hypothesis of this article was: Does the inclusion of CAM in basic health insurance reduce patient-related cost in Swiss primary care?

## Methods

The study was designed as a cross-sectional survey among primary care physicians providing conventional and/or complementary and alternative primary care in the five disciplines listed above. The eligibility criteria for inclusion in the study were defined as follows:

- Activity as primary care provider in a single-handed or group practice for at least two days per week.

- Holder of a CAM certificate approved by the Swiss Medical Association (CAM physicians) or no medical activity in CAM (COM physicians).

- Availability of reimbursement data for 2002 and 2003.

- No medical activity using exclusively acupuncture.

### Data collection, sampling procedures

A list of certified CAM physicians was acquired from the Swiss Medical Association in the year 2001. Membership lists of societies for complementary medicine (Swiss medical associations for homeopathy, anthroposophic medicine, neural therapy, and traditional Chinese medicine) also were obtained, and all CAM-certified physicians working as primary care physicians were asked to participate in the project. A list of all primary care providers (i.e. GPs, general internists) in Switzerland was additionally obtained from the Swiss Medical Association (FMH), from which a random sample of primary care providers not certified in any CAM discipline was selected and asked to participate. It was assumed that these physicians were less motivated to participate in the project. Therefore 1.5 times more non-CAM-certified physicians were sampled. This sample was proportionally matched to the regional distribution of physicians certified in complementary medicine.

Data collection was based on self-administered questionnaires and on reimbursement data available from the data pool of all Swiss health insurers (santésuisse) for 2002 and 2003. A questionnaire with accompanying letter explaining the purpose of the project was mailed to an initial sample of 2,266 physicians. The questionnaire covered professional qualifications, practice characteristics and self-declared activity in primary care and in CAM. Questionnaires were provided either in German, French or Italian, depending on mother tongue of physicians, and were mailed in summer 2002. Nonresponders obtained one reminding letter one month later. 812 questionnaires were initially returned (36%), 184 physicians refused participation (8.1%) and no answer was recorded for 1270 physicians (56%). 587 physicians met the inclusion criteria. Physicians not included were removed mainly because they were working in hospitals, providing acupuncture only, or working less than two days per week as general practitioners.

The reimbursement data, based on a fee-for-service framework, included expenditures covered by the mandatory, basic coverage offered by Swiss health insurers. These data were organized into direct consultation costs, and costs for medication either directly dispensed or prescribed to patients and into referral costs for laboratory analyses and physiotherapies. Cost from all other referrals (e.g. to consultants, imaging procedures or to hospitals) were not available.

Further data included demographic information of patients and patient load, i.e. the number of patients seen by each physician during one year. Reference data of all Swiss primary care providers including demographics and professional qualification were additionally obtained from the FMH.

Physicians were classified based on self-declared activity in CAM and on professional qualification into three groups:

- **COM physicians**: Physicians performing no CAM procedures (conventional primary medical care physicians).

- **Noncertified CAM physicians**: Physicians performing CAM and COM procedures without professional certification in CAM and without reimbursement of expenditures for CAM procedures in basic health insurance.

- **Certified CAM physicians**: Physicians performing CAM and COM procedures with CAM certificates provisionally recognized by basic health insurance (homeopathy, anthroposophic medicine, neural therapy, traditional Chinese medicine).

### Data analysis

Questionnaire data were recorded using standard database software and linked with the reimbursement data of each physician. The physician's identification code in the health insurers' database was used as unique identifier and for checking plausibility of data. Average reimbursements per patient were calculated based on total yearly costs generated by each physician and compared between groups using descriptive procedures in a first step. Linear models were used in a second step for statistical analysis of major cost components. The following data were analysed as major outcome variables with multiple models:

- Consultation related costs, i.e. all costs generated during a consultation (mainly related to the length of consultation)

- Referral costs (including lab analyses and physiotherapies only)

- Medication costs, including costs for medication directly dispensed to patients and prescribed medications.

- The sum of all costs (consultation related, medication, and referral costs).

All statistical models used in the study were equally structured in order to allow comparative analyses across the various cost components. The following covariables were included:

- Group: Certified- or noncertified CAM physicians, or COM physicians

- Canton: Geographic location of practice in Switzerland (26 Cantons)

- Urbanisation of practice location: central city, suburb, isolated town or village, rural area

- Type of practice: individual or group practice

- Gender of physician

- Experience of physician as years since graduation

- Proportion of consultations with female patients per year

- Average age of patients

- Proportion of home visits per year

- Proportion of accident related consultations per year

The selected covariables were based on preliminary univariate procedures. Colinearity and correlation among covariables was checked and appeared not to be a problem. Results were interpreted as least-square means (LS-Means) with 95% confidence intervals (CI95). Pairwise comparisons between groups were performed in case of significant overall differences and the Bonferroni procedure was used to adjust for the problem of multiple comparisons. Regression coefficients were used to estimate effect size of continuous co-variables. Residual analyses were applied to assess the fit between observed and modelled data, and multivariate power calculations were performed in case of nonsignificant differences. The amount of variance of outcomes accounted for by the models was expressed using R^2^-values. Two physicians with fivefold above average total cost were identified during these analyses (one COM and one noncertified CAM physician). The respective data were considered as outliers and removed from further assessment. Residual analysis of alternatively applied log-linear models showed a similar fit between observed and estimated data; data analyses were therefore based on the original data. The level of significance was set to 0.05 throughout the study and SAS 9.1 was used for calculations. All cost data are given in Swiss Francs (CHF) as of 2003.

## Results

The final sample comprised 262 certified CAM physicians, 151 noncertified CAM physicians and 172 COM physicians. Certified CAM physicians represented 40% of their registered base population. The number of noncertified CAM physicians and physicians performing exclusively COM are actually not known for Switzerland; the corresponding sampling proportions therefore cannot be calculated. However, 172 COM physicians represented 2.9% of all primary care providers listed by the FMH in 2002. Twenty-nine physicians were certified in anthroposophic medicine, 141 in homeopathy, 26 in neural-therapy and 94 in TCM. Twenty-eight physicians had multiple CAM certificates. No certification for phytotherapy was accredited by the FMH at the begining of the project; therefore no physicians with a respective certification were present in the study. The geographical distribution of sampled COM physicians by canton as well as their gender distribution was not significantly different from the respective distributions of all certified CAM physicians in the same year. Considerable and significant differences were observed between physicians for the self declared extent of medical activity in primary care. COM physicians declared 77.4% (median 90%) of their activity as primary care, non-certified CAM physicians 64.6% (75%) and certified CAM physicians only 36.8% (39%).

### Attributes of patients and consultations

The average ages of patients were significantly different between groups, 38.1, 43.2, and 47.3 years, for CAM certified physicians, noncertified CAM physicians, and COM physicians respectively. The proportion of respective consultations also differed significantly between groups. Female patients were seen in 66.8%, 62.1%, and 59.4% of consultations of certified, noncertified, and COM physicians respectively. Certified CAM physicians performed significantly fewer accident-related consultations (1.1% for certified, 2.4% for noncertified, and 1.8% for COM physicians) and made significantly fewer home visits to patients than noncertified CAM or COM physicians (1% for certified-, 3% for noncertified, and 4% for COM physicians).

### Health insurance expenditures

Certified CAM physicians obtained reimbursements for an average of 652 patients per year, whereas noncertified CAM physicians had 955 patients and COM physicians had 987 patients reimbursed; the differences between the groups were statistically significant. Average annual numbers of consultations and home visits were 2797 for certified CAM, 3810 for noncertified CAM and 3918 for COM physicians. Additional attributes of consultation are given in Table 1.

Neither part- or full-time activity of physicians nor patient demographics account for different average numbers of patients and consultations. An adjusted annual number of consultations per patient may therefore be more appropriate to assess the services provided. The adjusted numbers of consultations per patient per year differed significantly between groups (p < 0.001, power = 0.84, R^2 ^= 0.48): 4.52 (CI95: 4.19–4.85), 3.88 (3.55–4.21), and 3.68 (3.34–4.02) respectively for certified CAM, noncertified CAM, and COM physicians (empiric data in Table 1).

Means and medians of health insurance expenditures per patient are given in Table 2. These data indicate lower total cost, lower referral cost, and considerably lower cost for directly dispensed and prescribed medication for both groups of CAM physicians compared to COM physicians. In contrast, consultation costs for certified CAM physicians were higher.

There are important effects of cofactors on the magnitude of services and costs. The most important factors to be considered, concern differences in structural characteristics of practices and patient demographics. Structural characteristics of practices include practice location, practice type, consultation patterns, gender, and the number of years since graduation as a proxy for physicians' experience. Demographic differences of patients mainly entail differences in age and gender. Also, the Swiss health system is organized at the cantonal level, which results in 26 different reimbursement systems. Cofactor analysis of other explanatory variables than physicians group revealed a wide range of significant and potentially relevant associations. However they were beyond the immediate scope of this paper (see Table 4) and a further interpretation of these findings was therefore omitted.

The results of these models indicate that total per patient costs are equal between groups (p = 0.48, power>0.99, R^2 ^= 0.55). Compared to COM physicians, consultation-related costs are significantly higher in certified and noncertified CAM physicians (p < 0.001, power>0.99, R^2 ^= 0.30); no significant difference was observed between certified and noncertified CAM physicians. Total costs of medication (referred and directly dispensed) were also significantly different between groups (p < 0.001, power>0.99, R^2 ^= 0.64), Total medication was almost equal between COM and noncertified CAM physicians, but considerably lower in certified CAM physicians. Significant differences also were seen for cost arising from referrals, including reimbursements for lab analyses or physiotherapy (p = 0.01, power>0.99, R^2 ^= 0.29). Additional results of pairwise comparisons between groups are given in table 3.

**Figure 1:**
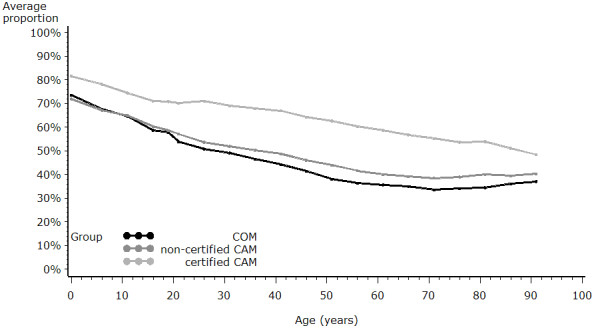
Proportion of consultation cost across physician groups and patient age.

Cost structure in terms of model-based percentages of total cost indicates that consultation-related costs account for 56.2% of costs in certified CAM physicians, 40.8% in noncertified CAM physicians and for 39.1% in COM physicians. These differences remained constant with reference to patient age (figure 1). Particular differences in cost structure were observed for costs related to medication when certified CAM physicians generated considerably lower relative costs than COM physicians (Table 3).

## Discussion

Health insurance expenditures for CAM were the subject of a long debate about the economics of CAM, which was characterized as much by ideological and political controversy as it was by a lack of valid data on costs and benefits in terms of patient-centred outcomes of CAM within Swiss primary care [3-5]. A further comparison with the international literature aimed at the economics of CAM therapies shows that research in this field is almost entirely restricted to specific indications and procedures[6], whereas studies adopting health system perspective are currently of low quality if not lacking at all[7]. The goal of this study was therefore to provide accurate information on the nature of CAM practice and its cost to social health insurance within the system of ambulatory care in Switzerland.

It is acknowledged in this context, that reimbursed cost do not necessarily reflect actual resource cost of care. However, the amount of socialized cost is a crucial component in maintaining and improving cost-efficiency and equity of health systems. The study focused on costs arising from interpersonal and medical-technical care, considered as proxies for consultation related priorities of patients and physicians [8]. Average costs per patient were placed at the centre of this study.

### Processes of care

The results confirm other observations that patients seeking CAM treatments are younger and more often tend to be female [9,10]. Furthermore, the study provides evidence of differences in process and management of care between CAM and COM. Certified CAM physicians treat fewer patients but spend more consultations and also more consultation time with those patients. They also treat fewer accident-related patients and perform fewer home visits than COM physicians. These differences, along with the low self declared activity in primary care of certified CAM physicians, have important implications for understanding CAM in Swiss primary care. CAM physicians care for a particular, distinctive selection of patients, and their consultation patterns may therefore not be fully in line with the formal definition of general practice/family medicine[11]. This obvious mismatch of defined and observed practice may adversely affect decisions on resource allocation and reimbursement policy for CAM.

### Extent of health insurance expenditures

Data on health care costs were obtained from the large data pool of all Swiss health insurers (santésuisse). These data are categorized into costs related to consultations, and referrals. Consultation-related costs cover almost 100% of all expenditures accounted for by the basic Swiss health insurance. Data on referral costs are restricted to prescriptions, lab analyses, and physiotherapy. The availability of cost data for other referrals – including hospitalisations, specialist treatments, and diagnostic or therapeutic procedures – is limited as only a minority of primary care providers act as gatekeepers. Hence respective data structures of insurers and health care providers do not allow a match for the remainders. Accordingly, expenditures on these procedures could not be compared in this study.

The modeling procedures of this study were based on a behavioural model in which predisposing factors such as beliefs and socio-demographic and behavioural attributes of patients indirectly influence health care use via patients' expectations and direct medical needs[12]. Health of patients was therefore regarded as an intrinsic component of providing and consuming care within a specific treatment philosophy, i.e. particularly CAM patients have specific procedures in mind when they decide to consult a physician. It was therefore not deemed appropriate to model resource utilisation as a function of CAM or COM by additionally controlling for health status of patients, although such data would have been available for a subsample of physicians[13].

Regardless of these limitations, our data on total annual treatment costs per patient show substantial differences between CAM and COM. Certified CAM physicians appeared to generate the lowest and COM physicians the highest costs to social health insurance. However, modelling procedures indicate significant confounding effects of some health system and patient-related cofactors. Differences between groups decreased considerably after incorporating these factors into the analyses. Consequently, total treatment costs per patient do not significantly differ, statistically, between groups. Statistical tests and effect sizes indicated significant and particularly large effects of patient age, patient gender, and the frequency of accident-related consultations as a proxy indicator for a more somatically oriented consultation style (Table 4). Hence these three factors are important predictors of providing and consuming CAM in primary care. Our study therefore provides evidence that apparent lower treatment costs of CAM in Swiss primary care are mainly related to structural attributes of care and to a more cost effective patient population; in other words, CAM physicians treat younger patients and have a larger proportion of less costly consultations with female patients. We have to assume in this context that female patients are seeking more often specialist care for cost intensive health problems than male patients and have therefore less costly individual consultations in primary care. This phenomenon, that younger, better-educated patients who have diseases of longer duration but slightly better overall health status are more prevalent in specialist practices than in generalist practices is well know in the literature[14]. Our finding are also in line with other studies within the same project evaluating CAM in Swiss primary care indicating that CAM patients utilize more frequent and more diverse medical services than COM patients[13]. It therefore remains doubtful that including CAM in basic health coverage would have had a long term, cost containing effect, on overall expenditures in Swiss health care. We also doubt that an inclusion of CAM in basic health insurance will be cost neutral. Firstly because every new procedure in the catalogue of reimbursed health services will add to the overall cost of the system per se. Additionally, the literature and earlier work in the context of this project suggest that CAM is not always a substitute for orthodox care and may be an additional expense.

### Structure of health insurance expenditures

The observed differences in cost structure between CAM and COM are a direct reflection of different philosophies of care in complementary and alternative, and conventional medicine. CAM physicians claim to pursue a more patient-centred and holistic approach that focuses on patient empowerment and self-healing, rather than just applying the biomedical model to cope with or to cure a specific somatic disease. The concept of patient centredness is attributed to the work of Michael Balint[15], who used the concept as related to illness-centredness. The concept is, however, closely related to humanistic psychology and the person-centred therapy originally developed by Carl Rogers in the 1940-ies[16]. Although patient centredness, despite being described as clinical method by Levenstein et al. [17], still remains difficult to define [18], the quality of the interaction between patients and their physicians is considered to be a major component of patient centredness [19]. Patient-centred care also entails partnership and a focus beyond specific disease conditions [20,21], which are key elements of care in the philosophy of both family medicine [22] and CAM. Furthermore, the literature indicates that the relationship between prescribing and direct consultation costs is associated with various structural and physician-related factors [23,24]. Prescription patterns are also related to the duration of consultations [23,25]: longer consultation time is associated with higher patient satisfaction, better patient enablement, and fewer prescriptions [26]. Our data on cost structure document the direct financial consequences of differences in the way care is provided by CAM and COM physicians. The data also support allegations made by CAM advocates that conventional physicians mostly rely, irrespective of supporting evidence, on medications provided directly by the pharmaceutical industry. But it is not possible, at least based on these analyses, to conclude that this particular split of health care spending is associated with better outcomes in primary care.

### Limitations and strengths

CAM procedures were defined within the legal framework of the Swiss health care system that included only homeopathy, anthroposophical medicine, neural therapy and traditional Chinese herbal medicine provided by physicians trained in both conventional and complementary primary care. As part of a project to evaluate the entire system of CAM provision in primary care in Switzerland, this study was not a controlled experiment. Selection bias and systematic differences that are not related to specific treatment philosophies were therefore unavoidable. It can be assumed that the motivation differed between participating physicians, since CAM physicians were under more pressure to demonstrate effective methods – which was not the case for COM physicians. It can only be speculated that the motivation of COM physicians is more attributable to a general interest in primary care research. In a strict sense, the generalisability of our results is therefore reduced to physicians with these distinct motivations. However, a comparison of the sample population with the general population of all Swiss primary care providers indicated no difference with reference to geographic location of practices and gender of physicians, clinical data of the project including patient perceived health status with regard to other recent research in Swiss primary care showed also no difference[13]. Therefore, and regardless of the low sampling fraction, we consider the study sample as a reasonable representation of Swiss primary care. Further problems are associated with aggregated patient attributes at the practice level that were used to adjust for effects of cofactors. The data provide therefore no possibility to track consumption patterns of individual patients within the entire health system. The most severe limitations are related to the different case mix of patients treated by CAM and COM physicians that could not be accounted for in the analysis. Other data within the project show that our sample of COM physicians treated more cardiovascular conditions entailing higher treatment costs, whereas CAM physicians treated more psychiatric conditions [13] that are generally associated with lower costs per consultation. While there is no indication for bias in favour of COM, it remains; however, open to which extent these findings result in biased pro CAM estimates of annual patient costs because psychiatric conditions tend to require more consultations than cardiovascular problems which may partly or fully compensate differences of consultation cost. Nevertheless, caution is advised when interpreting differences of average reimbursements per patient between CAM and COM. Further limitations apply also to the fact that only costs for medication, referred laboratory analyses and physiotherapies are included in the health insurers data pool. No data are therefore available for costs of hospitalisations, of expensive diagnostic procedures such as MRI- or CT-scans and for referrals to other physicians and specialists.

Among the strengths of the study are the complete billing data of participating physicians, and, in contrast to other research [27], a considerable amount of variance in cost outcomes could be explained by statistical models used in the study. Most models had enough power to identify various statistically significant differences, or their lack, in health care expenditures between CAM and COM.

Additional research within the scope of this project will provide more information on health status of patients as seen by physicians and fulfilment of patient expectations. Further analyses will also investigate the relationships between use of resources, patient satisfaction, and treatment cost.

## Conclusion

Our data provide little evidence that patient-related costs of CAM are lower than costs in conventional primary care. However, if direct consultation costs are taken as a proxy indicator of interpersonal care, CAM appears to deliver more interpersonal primary care than COM. Hence, if strategies of patient empowerment and patient-centred care are to be pursued in the future, the results of the study can be used as a guideline to improve primary care in order to achieve its overall goal of providing highly effective interpersonal care. This has implications for resource allocation – especially for resources related to the time physicians interact with their patients, but also resources related to the education of future primary care physicians. In fact, consultation time may be seen as a "common currency" of high quality primary care [28]. But crucial questions remain about the use of CAM by different populations. The decision of the Swiss federal health authorities to exclude CAM from basic health insurance therefore remains debatable. This decision indicates that the importance of patient-centred primary care is not yet fully recognized by the Swiss health system despite the fact that self empowerment and patient-centred approaches are promoted by the Swiss Academy of Medical Science and other institutions [29,30].

## Competing interests

The Swiss Federal Office of Public Health funded the project and by contract researchers were independent from the funder. The authors declare that they have no competing interests.

## Authors' contributions

AB was the principle investigator of the field studies of the project; he performed all statistical analyses and wrote the first draft of the manuscript. RE designed the economical analyses and reviewed and supplemented the manuscript in this context. BK provided substantial input with reference to all aspects of primary care and reviewed and supplemented the manuscript.

## Pre-publication history

The pre-publication history for this paper can be accessed here:



## Figures and Tables

**Table 1:** Empric means, medians of patients and consultations. (data from health insurance records of individual physicians)

	certified CAM		noncertified CAM		COM	
	mean	median	mean	median	mean	median
Number of patients per year	652	537	955	899	987	906
Number of consultations per year	2797	2440	3811	3726	3918	3783
- Disease related	2746	2421	3694	3621	3822	3731
- Accident related	40	14	100	76	82	46
- Other (maternity, unclassified)	12	2	17	3	13	2
- Number of home visits	79	30	268	181	304	211
Number of consultations per patient and year	4.6	4.2	4.1	3.9	4.2	4.0

**Table 2:** Reimbursements per patient in Swiss Francs

Type of reimbursement	certified CAM		noncertified CAM		COM	
	mean	median	mean	median	mean	median
**Consultation related costs**	**428**	**367**	**319**	**304**	**334**	**315**
**Referral costs**	**67**	**55**	**91**	**77**	**98**	**89**
- Lab analyses	31	23	40	27	42	33
- Physiotherapy	35	27	51	43	56	53
**Total costs for medication **(directly dispensed & referred)	**198**	**142**	**384**	**351**	**484**	**458**
- Costs of dispensed medication	71	29	159	92	145	46
- Prescriptions	130	60	226	106	337	278

Total costs	**691**	**630**	**794**	**754**	**911**	**878**

**Table 3:** Model based means (LS-means) and structure of reimbursements per patient in Swiss Francs

Type of reimbursement	certified CAM			noncertified CAM			COM		
	LS-mean	CI 95	%	LS-mean	CI 95	%	LS-mean	CI 95	%
Consultation related costs*	**408^**a**^**	362–455	56.2	**304^**b**^**	258–350	40.8	**278^**b**^**	231–326	39.1
Referral costs*	**63^**a**^**	47–78	8.7	**82^**b**^**	67–97	11.0	**76^**ab**^**	60–91	10.7
- Lab analyses*	25^a^	16–34	3.4	39^b^	30–47	5.2	35^b^	26–44	4.9
- Physiotherapy	37	28–47	5.1	43	33–52	5.8	40	30–50	5.6
Total costs for medication* (directly dispensed & referred)	**255^**a**^**	213–297	35.1	**362^**b**^**	320–404	48.5	**362^**b**^**	319–405	50.9
- Costs of dispensed medication*	96^a^	68–125	13.2	135^b^	107–163	18.1	132^b^	103–160	18.6
- Prescriptions	161^a^	115–207	22.2	227^b^	181–272	30.4	228^b^	181–275	32.1

Total costs^†^	**726**	661–792	100.0	**746**	681–811	100.0	**711**	644–778	100.0

* Significant overall difference between groups (p < 0.05).

^% ^Percentage of total cost per patient.

^a, b, c ^Different superscripts indicate significant differences between groups (multivariate linear models).

^† ^LS-means of total cost are not the exact sum of estimates of cost components.

**Table 4:** Effects of factors of major cost components

Factor	Parameter estimates of continous data^a^			
	Consultation related cost	Referral related cost	Total cost for medication	Total costs
Experience of physician in years	-0.26	-0.31	1.69*	1.24
Proportion of consultations for female patients	-4.18*	0.31	-5.88*	-9.71*
Average age of patients	6.48*	1.86*	12.72*	21.24*
Proportion of home visits	-793.01*	178.68*	546.16*	-16.45
Proportion of accident consultations	-2718.22*	13.15	-581.06	-3165.41*

	Overall F-tests of categorical variables^b^			
	p-value	p-value	p-value	p-value

Physicians group	<.001	0.011	<.001	0.483
Canton	0.020	0.001	<.001	<.001
Practice location	0.286	0.006	0.354	0.057
Type of practice	0.025	0.881	0.492	0.065
Gender of physician	0.467	0.706	0.004	0.009

^a ^interpreted as change of cost per change for each unit of the respective factor (years and percentages).

^b ^interpreted as differences of least square means of the respective factors.

* Significant association (p < 0.05)
